# Capitalism, COVID‐19 and lockdowns

**DOI:** 10.1111/beer.12431

**Published:** 2022-04-09

**Authors:** Philipp Bagus, José Antonio Peña‐Ramos, Antonio Sánchez‐Bayón

**Affiliations:** ^1^ Faculty of Social Sciences, Department of Applied Economics I and History and Economic Institutions and Moral Philosophy King Juan Carlos University Madrid Spain; ^2^ Faculty of Social Sciences and Humanities Universidad Autónoma de Chile Providencia Chile; ^3^ Faculty of Social Sciences Economía de la Empresa (ADO), Economía Aplicada II y Fundamentos Análisis Económico Madrid Spain

**Keywords:** capitalism, COVID‐19, epidemics, libertarianism, lockdowns, property rights ethics

## Abstract

Commentators believe that the COVID‐19 pandemic reveals the inconveniences of capitalism and that the end of “neoliberalism” could be near. In this article we show that a capitalist ethics is capable to deal with the challenges of pandemics and comes with important advantages such as the prevention of overreactions. We apply both utilitarian and rights‐based ethics to the case of epidemics in general and COVID‐19 in particular. First a libertarian natural law ethics is used to assess the government interventions in the Corona pandemic. We maintain that these interventions cannot be justified from a libertarian point of view despite of the possible objections that are discussed such as the “potential threat argument”. Moreover, the utilitarian argument in favor of government lockdowns is evaluated. The negative effects of lockdown on mental health, addictions, domestic violence, etc. have to be taken into account. The utilitarian argument in favor of lockdown is far from convincing, as economic calculation is not possible.

## 1 INTRODUCTION

As a response to the COVID‐19 epidemic, governments have infringed upon private property rights (and human rights) to an unprecedented degree in peace times. They have expropriated and confiscated medical equipment and material from businesses, they have taken control of private health companies and hospitals, they have decreed the forced closure of private businesses, such as private kindergartens, schools, universities, restaurants, hotels, or retail stores. Governments have even ordered the closure of private parks and gardens. Moreover, they have severely restricted the freedom of movement.

While the economic and social consequences of the lockdowns are undisputed, their ethicality must still be revised. The following questions abound in case of epidemics: Is the government justified using force in order to shut down businesses and effectively make it impossible for millions of people to go to work, thereby affectively preventing a large part of the capitalist system doing its work? Moreover, is it justified to confine people, effectively depriving many businesses from their customers and workers from the chance of earning a living?

In the public debate, there is consensus that the state should intervene and restrict human rights in an emergency such as the Corona epidemic. The argument, which can be found, for instance, in Gavin (2020), Branswell (2020), Baldwin and Taghipour (2020) or Salt (2020), may be summarized as follows: (1) The life of human beings is the greatest good and has to be protected.^1^ (2) The capacity of the health care systems is limited. (3) A rapid infection of the whole population would lead to more deaths than a slower infection due to the limited capacity of health care systems. (4) Only strict social distancing can most effectively “flatten the curve” of infections. (5) Therefore, the state is justified in enforcing social distancing and restricting human rights such as freedom of movement, because it saves human lives.

Some commentators believe that the COVD‐19 epidemic shows the inconveniences of the market (Klingenberg, 2020; Masan, 2020; O´Mara, 2020) and believe that the end “neoliberalism” could be near (Delanty, 2020). The argument goes that a capitalist system cannot deal appropriately with pandemics.

For example, Klingenberg (2020) states that “[w]e’re now seeing the market‐based models for social organization fail, catastrophically, as self‐seeking behavior (from Trump down) makes this crisis so much more dangerous than it needed to be.”

No one less than the Pope remarked in his encyclical letter *Fratelli Tutti* that “[t]he fragility of world systems in the face of the pandemic has demonstrated that not everything can be resolved by market freedom.” (Pope Francis, 2020).

Libertarian approaches to epidemics are considered to be inadequate (Delanty, 2020; Koehler, 2021), as the need of a strong powerful state is considered to be self‐evident in the wake of the COVID‐19 (Oreskes, 2020; The Economist, 2020). In the same vein, the influential philosopher Žižek (2020) regards the pandemic resulting from the virus of capitalism and an opportunity to reinvent communism. More moderately, Delanty (2020) believes that neoliberalism is dead, and that we may come out of the crisis with a more humanized form of capitalism.^2^

In this article it is analyzed if the pandemic really shows inherent problems of the free market and capitalist ideology. Is libertarianism unable to confront such vital questions as pandemics?

Our main contribution is to show that a capitalist ethics is capable to deal with the challenges of pandemics and comes with important advantages such as the prevention of overreactions. In our analysis, we focus on two ethical approaches that are commonly defended by libertarians in order to defend the capitalistic order, namely utilitarianism and natural law ethics. Utilitarian defenses of capitalism argue capitalism leads to peace and prosperity, examples being Mill (1859), von Mises (1998) or Friedman (1973). Natural law defenses of capitalism focus on property rights and self‐ownership as the ethical foundation of capitalism, examples being Rand (1967), Rothbard (1982) or Machan (2002), We do not focus on alternative approaches such as the Rawlsian view of justice or a Kantian duty approach, even though one could make a case against general lockdowns based on these approaches, since they are not been used as frequently for a defense of capitalism as utilitarianism or natural law ethics.

First, the article spells out the libertarian natural law theory as most coherently exposed by Murray Rothbard in his seminal work *The Ethics of Liberty* (1982), in which he develops an ethic of capitalism. In the following the reasoning behind libertarian natural law ethics is presented and the four basic rules of a private property or purely capitalistic society are portrayed. Then the libertarian reasoning is applied to ask: What can be said about the government restrictions of liberties in the COVID‐19 pandemic from a libertarian point of view? Can they be justified? We then show that the even the classical liberal John Stuart Mill comes to similar conclusions as libertarian property rights ethics.

Finally, the utilitarian argument in favor of these measures is examined. The argument is made that the utilitarian verdict in favor of government interventions is not clear‐cut and that it does not consider the problems of economic calculation.

## 2 PRIVATE PROPERTY RIGHTS ETHICS

Rothbard (1982) follows the traditional medieval natural law philosophers such as Suárez (2012) and Grotius (2018). According to these authors, natural law is objective in the sense that all human beings can use reason to deduce these natural laws. In the same sense that it is in the nature of an apple to fall to the ground, there are also things that are in the nature of man. Natural law theory maintains that through rational reasoning one can detect these things.

Libertarian natural law theory is based on the writings of Locke (2018), Spencer (1970), Spooner (1973), and the American Declaration of Independence (1776). A crucial point in natural law ethics is to distinguish between natural rights and the morality or esthetics of exercising these rights. Ethics, according to Rothbard, is the doctrine of the legitimate use of violence: When is someone allowed to use violence against another person? For instance, someone might not want to greet a neighbor on the street. According to libertarian ethics, no one has the right to use violence to force people to be polite and greet their neighbors, by sending them to prison if they fail to do so. However, one could make the argument that it is impolite not to greet. One could even make the argument that it is immoral not to greet or spit to the ground as it amounts to an insult. Rothbard separates the question of morality (such as politeness) from ethics. He is concerned only with ethics, that is the question when physical violence, or the threat thereof, is justified.

### 2.1 Rothbard’s four rules of private property

Libertarian natural law ethics will now be presented briefly. Rothbard (1982) carefully deduces four rules for his private property natural law ethics. First, everyone is the sole owner of his or her own body. That is the principle of self‐ownership. Second, everyone becomes the rightful owner of hitherto unowned resources that he or she starts to use. That is the homesteading or finder keeper principle. Third, everyone is the rightful owner of the things he produces with his own labor and resources. That is the production principle. Fourth, everyone is the rightful owner of the things he exchanges voluntarily with other rightful owners, including the gifts he receives. That is the exchange principle. Liberty is alive when property rights are not violated. One is not allowed to uninvitedly harm the property and life of others. The four rules imply the basic human rights to property and to life. Infringements on private property rights are defined as crime.

Natural law ethics is universal, which means that it is independent to time and place and applies to all human beings. In other words, it also applies in times of emergencies such as epidemics.

Rothbard (1982) justifies his four rules the following way. For the first rule he asks: what are the alternatives toward self‐ownership? He finds two alternatives. The first one is that the body of person A is the property of person B. This alternative rule violates the universality principle, since some people are the property of others. There are slaves and masters. Different laws apply to slaves and masters; they are not universal for all. The master can do whatever he wants with his body and the slave’s body, while the slave cannot do whatever he wants with his body and the master’s body. Hence, the first alternative does not qualify as a universal ethics. The second option is that all human beings are the co‐property of all other human beings. This second alternative is not functional, because before we could do anything with our body, we would need the permission of everybody else (all co‐owners), but these owners could not give the permission without asking for permission to do so. And so on. The alternative of “universal communism” implies that mankind would die out. Therefore, the rule of self‐ownership is the only viable and universal option.

As for the justification of the second rule, Rothbard follows the reasoning of Locke (2018). When we mix our ideas, work and nature given resources, they become our property. We can homestead unowned resources and become their rightful owners. The alternatives according to Rothbard are analogous to rule one. First, the resources someone homesteads could become the property of another person, but this would violate again the universality principle. This is so, because some people (the masters) could homestead unowned resources and use them, while others (the slaves) would have to transfer the homesteaded resources to others (the masters). Second, the homesteaded resources could become property of whole humanity. Then we run again into the problem that we had to ask for permission of everyone else if we wanted to use a resource. Thus, we are left with the option that by homesteading natural given resources become legitimate property. The justification of the third and fourth rule can be deduced from the first two rules. By producing an owner is mixing his property with his labor. As it is his property, he can exchange it voluntarily against the property of someone else or give it to him. This is (Rothbardian) libertarian natural law ethics in nutshell.^3^

Any violation of the four rules is a crime and the legitimate owner has the right to defend himself against the aggression (in a proportional manner). In other words, the only instance when physical violence is legitimate is to defend one’s property rights. This principle is also known as the nonaggression principle. The initiation of violence is inherently wrong. As Block (2003) puts it:

It shall be legal for anyone to do anything he wants, provided only that he does not initiate (or threaten) violence against the person or legitimately owned property of another.^4^

## 3 LIBERTARIAN NATURAL LAW AND COVID‐19

After having briefly exposed the ethical principles of libertarianism, this framework is now applied to assess the restrictions of liberties undertaken by governments during the COVID‐19 crisis with a few exceptions such as Sweden.

To being with, are governments justified to restrict the freedom of movement in order to slow down the infection rate?

It could be argued that most streets are government property, and that the government has the right to restrict freedom of movement on its streets in order to protect the health of its citizens. Indeed, the public ownership of streets is a problem from a libertarian perspective. Streets should be private. If streets were private, the owners would decide who could use them and under what conditions. As Rothbard puts it:

In the libertarian society…streets would all be privately owned, the entire conflict could be resolved without violating anyone’s property rights: for then the owners of the streets would have the right to decide who shall have access to those streets, and they could then keep out “undesirables” [in our case people suspected of being infected with viruses] if they so wished. (1982, p. 119)

In other words, in a libertarian or purely capitalist world private street owners would decide which streets would remain open, to whom, and under what conditions in an epidemic.

Yet, we live in a world where the majority of streets are public. However, even with public streets Rothbard’s verdict is clear. Discussing the case of a McDonald’s restaurant opening and residents protesting the gathering of its customers on the streets, Rothbard writes:

as taxpayers and citizens, these “undesirables” [the customers] surely have the “right” to walk on the streets, and of course they *could* gather on the spot, if they so desired, without the attraction of McDonald’s. (1982, p. 119)

In Rothbard’s view, citizens and taxpayers have the right to use public streets. Governments are not justified in restricting movement on their streets, because in fact the street is not even the just property of the state. The state has no right to determine who can use public streets and who cannot. A confinement or curfew are violations of private property rights and cannot be justified.

In a libertarian world with private streets and private businesses, the owners impose the rules. In the same way, that private street owners could enforce safety standards, license plates, mandatory seat belts, alcohol limits, or speed limits for care, they could enforce mandatory face masks or distancing for pedestrians. In the case of an epidemic, business owners may close their property completely to the public. Or they could invite people conditionally to their property. For instance, they could limit the number of people who can access it. They could require tests before entering the property or declare that entering is at their own risk. They could also impose certain conditions, such as an age restriction or the required wearing of masks and gloves. This decision would be based on the personal moral deliberations of the business owner.

Let us discuss the other restrictions that have been implemented in the wake of the COVID‐19 epidemic, such as the required closing of bars, hotels, and other businesses. The politicians' argument in favor of the closures is the following: out of solidarity with the rest of the population, especially with the elderly, people should help bring the rate of infection down, because otherwise many people will die due to the limited capacities of the public health systems and the lack of provision for such an epidemic. People staying at home, confined to their houses, would save lives. They would thereby help others. And as people cannot be expected to help others and stay at home voluntarily, the state has the right to enforce a confinement that saves lives.

Now, the essential ethical question is the following: is anyone allowed to use violence in order to ensure that people will help their fellow men? Can the use of coercion to make people help others be justified?^5^

Rothbard’s answer to this question in *The Ethics of Liberty* is unequivocal:

it is impermissible to interpret the term “right to life,” to give one an enforceable claim to the action of someone else to sustain that life. In our terminology, such a claim would be an impermissible violation of the other person’s right of self‐ownership. (1982, p. 99)

Note that for Rothbard and libertarians in general, the concept of “rights” is purely negative. For instance, there is no right to a reasonable standard of living. Rights protect the radius of a person’s action that no one else can interfere with using aggressive violence. Property rights demarcate the area in which an individual can act freely.

Rothbard continues:

No man can therefore have a “right” to compel someone to do a positive act, for in that case the compulsion violates the right of person or property of the individual being coerced…. As a corollary, this means that, in the free society, no man may be saddled with the legal obligation to do anything for another, since that would invade the former’s rights; the only legal obligation one man has to another is to respect the other man’s rights. (1982, p. 99)

Rothbard gives two examples to argue that no one may use violence to make someone help another person. First, he discusses an example provided by von Hayek (1960, p. 136). In this example there exists a “monopolist” owner of water in an oasis. Rothbard (1982, p. 221) points out that the owner has the right *not* to sell the water to customers. The owner is within his rights in reserving the water for himself and cannot be forced to help thirsty people by selling the water.

Rothbard provides a second example for his claim that no one can be forced to help others. This example is about an *epidemic* and, therefore, is worth quoting in full:

Suppose that there is only one physician in a community, and an epidemic breaks out; only *he* can save the lives of numerous fellow‐citizens—an action surely crucial to their existence. Is he “coercing” them if (a) he refuses to do anything, or leave town; or (b) if he charges a very high price for his curative services? Certainly not. There is, for one thing, nothing wrong with a man charging the value of his services to his customers, i.e., what they are willing to pay. He further has every right to refuse to do anything. While he may perhaps be criticized morally or aesthetically, as a self‐owner of his own body he has every right to refuse to cure or to do so at a high price; to say that he is being “coercive” is furthermore to imply that it is proper and *not* coercive for his customers or their agents to *force* the physician to treat them: in short, to justify his enslavement. But surely enslavement, compulsory labor, must be considered “coercive” in any sensible meaning of the term. (1982, p. 222)

If the physician cannot be forced to help during an epidemic, then *a fortiori* a normal citizen cannot be forced to help either. It is certainly possible that one could help others in these times by staying home, by closing businesses, or by donating medical equipment. Yet forcing people to stay at home, closing their businesses, and expropriating medical equipment are violations of property rights.

The owner of a business has the right to open it. The workers have a right to go to their jobs. The owner of a garden has the right to use it and the pedestrian has the right to walk on the street. They are only responsible for their own actions and their own property and not for the existence of the coronavirus or potentially overwhelmed public hospitals.^6^

Of course, it is a different case if someone knows that he is infected and opens his business with the intention of infecting and doing harm to the customers. This would be criminal behavior and defensive violence, such as closing down the business by the threat of force, would be justified. But how does one know that the opening of the business is really an act of aggression on part of an infected owner?

Rothbard (1982, p. 78) points out, that the burden of proof is on the people using violence. No one is justified in using violence just because he perceives some risk of a potential threat. The threat must be proven in court. The threat of aggression must be “palpable, immediate, and direct.” It does not suffice to say that I feel threatened by people walking on the street because they might infect me. Someone can always perceive risk and a potential threat. Similarly, I might state that I feel threatened by cars driving on the street because they might hit me. However, that does not give me the right to use violence and stop others driving their cars. First, I would have to prove that they intentionally want to do harm with it (for instance, they are plotting a terrorist attack with a truck). Or I would have to prove that another person is negligent, for instance, driving drunk. Applied to our case, I would have to prove that someone intentionally wants to infect others or that someone is infected *and* does not hold sufficient distance from others. Appealing to a possible risk of infection alone is not sufficient. Indeed, allowing violence in case of perceive risk gives leeway to a war of all against all. As Rothbard puts it:

Once one can use force against someone because of his “risky” activities, the sky is the limit, and there is virtually no limit to aggression against the rights of others. Once permit someone’s “fear” of the “risky” activities of others to lead to coercive action, then *any* tyranny becomes justified … (Rothbard, 1982, pp. 238–239).

In this context, the legal principal of *in dubio pro reo* is vital.^7^ One only knows if someone is a criminal when he is convicted. Until people are convicted, they must enjoy all the rights of innocents, such as being allowed to leave their houses or open their stores. As Rothbard (1982, p. 82) reminds us, “they are innocent until proven guilty.”

### 3.1 COVID‐19 and preemptive violence

Kluger (2020) and Gander (2020) maintain that it is unethical to leave one’s house during the corona epidemic. Their argument is that by leaving one’s house one could contribute to the transmission of a deadly virus. There are also libertarians such as Olson (2020) or Shapiro (2020), and, more hesitatingly Huemer (2020) who, following a right‐based ethics, make the case for confinement. According to these pro‐capitalistic authors, people can be confined during a pandemic such as the present one, because they are (potential) aggressors. Anyone could unknowingly carry the virus and transmit it, and therefore poses a potential threat to the health of others.

The argument of preemptive violence as a justification for lockdowns is strong and seems to be compatible with libertarian natural rights ethics. However, preemptive violence cannot be justified against someone who is just a *potential* aggressor. One must prove beyond any reasonable doubt that someone is infected and wants to infect others or behaves negligent. It is unethical to use defensive violence without having proven an imminent attack.

In this context, Rothbard (1982, p. 82) argues that a policeman can use coercion against a suspected criminal on his own risk. If the suspect finally is found to be innocent, the policeman must be treated as a criminal. On the contrary, the policeman can only be exonerated if the suspect finally is proved to be a criminal. One complicating issue is that a policeman may not have the time to know if the threat is real. Let us take the example of a policeman who uses violence against someone who threatens him with a dummy weapon. The policeman does not know that it is a dummy weapon and could be exonerated later.^8^ Similarly, the government may not know if people are infected, in which ways a virus spreads and how dangerous it really is. Therefore, one might argue that governments can use coercion confining people and could be exonerated as it does not have enough knowledge about the danger of the virus. Indeed, this argument that regards lockdowns as protection of property rights in times of extreme uncertainty about the real threat is well thought.

However, there are several problems with the dummy gun analogy. First, there is no one actively threatening in the case of an epidemic as is the case of the dummy gun. Second, in both cases a means is required that is proportional to the end sought (Rothbard, 1982, p. 80). Confining someone may not be justified as other less invasive means such as social distancing are available. Third, while in the case of the dummy gun, a reasonable man would regard it as an imminent threat, the case of a potential virus is different. The probability of being infected with a new, deadly virus that can infect other people through the air via long distances is low, especially in the outside. Fourth, there is not much time to think in the case of the gun dummy threat, while in the virus case, there is time to reason and think. And as long there is no strict evidence that there is an imminent threat violence cannot be initiated. Again, the burden of proof is on the person using defensive violence. In other words, if someone does not know beyond reasonable doubt, that the other person has a virus that spreads easily through air over long distances and is very dangerous, violence is not permitted. Non‐knowledge or ignorance does not excuse violence. If the policeman decides to intervene, it is on his own risk. If the suspect is innocent, the policeman is punishable for the violation of property rights. Fifth, the dummy gun holder may actually apply the analogy in his favor. The gun holder may argue that he just wanted to confine the policeman as he thought that the policeman could be infected with a deadly virus.^9^ As the policeman was a potential threat, the dummy gun holder was justified in threatening him. There is no limit to violence of all against all once one allows for it without having to prove that there was the imminent threat of an attack.

Naturally, one must distinguish intentional attacks from negligence. Hoppe (2004) has criticized Rothbard for only focusing on causality and not on fault that is intentionality and negligence. Hoppe (2004, pp. 89–90) argues that beside intentionality and negligence there exists also faultless causation:

[F]aultless causation, which remains free of punishment, exists also. Life involves an inescapable element of risk. It is incumbent on each individual to learn how to live with such risk and to insure himself against it. However, this implies admitting that the narrow causality criterion is inadequate. What needs to be added to Rothbard’s criterion would seem to be this: No one is liable for “accidents” involving his person and property. Instead, the risk of accidents and the insurance against them must be assumed individually (by each person and property owner for himself). People can be held liable only for their actions, whether intentional or negligent (but not for accidents involving them).

In other words, one has to distinguish between negligent behavior and accidents. It seems reasonable not to allow negligent behavior when it endangers the private property of others, such as other street users on a public street. For example, a drunk driver who is not in control of his car and does not keep a prudent distance could be considered to be acting negligently, and it seems to be justified to pull this driver over. Applying this reasoning to the corona epidemic, someone who is infected with the virus and does not keep far enough away from others or sneezes in the street without covering could be asked to take precautions or be sent back to his property. What is clearly unjust and disproportionate is to prohibit everyone from driving because of the mere possibility of negligent driving, or to quarantine everyone because there is a risk of infection.

For a crime to exist from a libertarian perspective there must be proven fault. If person A infects person B unintentionally and unknowingly with a cold, a flu or COVID‐19, this must be considered to be an accident. Person A cannot be held liable. Again, the case is different if person A intentionally infects person B, for instance, by secretly spitting in his tea cup. Similarly, A would be liable if he acts negligently sneezing person B in her face without covering his mouth.

To make the point more pronounced let us apply this reasoning to the following example: imagine someone aiming a deadly arrow at a tree on his property. The assumption is that if he misses his target, the arrow will fly into his neighbor’s property, possibly hurting innocent people. Is shooting the arrow negligent behavior that should be stopped? Depending on the exact circumstances, so it seems. However, it does not justify quarantining the general population. First of all, not everyone owns a bow and arrow. Similarly, today not everyone has the virus and can “shoot” it at others. Second, not everyone who owns a “bow and arrow” (is infected) “shoots” (spreads germs) negligently in the direction of their neighbors.

If confining everyone because they could become infected, and in addition could act negligently, is justified in the case of the epidemic, one can make an analogous argument in the case of the bow and arrow: anyone could in principle purchase a bow and arrow and could in principle shoot arrows negligently. Hence, anyone is a potential “arrow threat” and everyone must be locked up in their homes. Or, alternatively, the sale of bows and arrows must be prohibited. However, this clearly violates private property rights. The violators of private property rights, far from occupying the moral high ground, must be considered criminals.

If one allows the use of violence against innocent people because they are a potential risk or threat to others, then there will be virtually no limit to the coercion that can be justified. For instance, in World War II the US government interned Japanese people and US citizens of Japanese ancestry in concentration camps, because these citizens were assumed to be a threat (McMaken, 2015). They were expected to commit acts of sabotage killing innocent people. Possibly these acts of sabotage would encourage others to copy these acts, leading exponentially to more and more acts of sabotage and the loss of the war with millions of deaths in the US. Even if one grants that there was a higher risk that US citizens of Japanese ancestry would commit such acts, this does not justify the internment of innocent people. One must prove that someone is planning to commit an act of sabotage. The proof must be presented for each individual. Responsibility is individual, never collective.^10^ If one allows violence based on collective guilt, there is no limit to violence.

Why not, as a preemptive measure, lock up ethnic groups that have had a higher probability of committing crimes in the past than other groups? When one allows violence against someone who is considered a threat based on statistics, the sky is the limit. And what about other infectious diseases? If infecting others with the coronavirus is an aggressive act, what about infecting others with the flu or a mild cold. A mild cold can develop into a severe problem for someone with a weak immune system. These are just differences of degree. If one of these instances is an aggression and immoral, the others are, too. What is a fair punishment for someone who spreads a cold? Shall the whole population be quarantined every winter because thousands die from the flu?

Why not confine the whole population all the time? It saves lives (or could save at least some lives in the short run). There always exists a risk that someone will catch a new, unknown virus, let us say COVID‐21, and will infect others, becoming an “aggressor.” Following this reasoning, anyone is a potential threat to anyone else—just by being alive and in contact with others, because he may spread bacteria and viruses.

Human beings live in nature and with things that they do not control completely. Unfortunately, accidents occur. Life is risky. Let us suppose that a driver’s tire was punctured on the highway, leading to an accident that hurt others. The person was a careful driver whose car had recently passed inspection. What happened was not due to the driver’s negligence, but an accident. It certainly does not justify prohibiting all driving. Is the transmission of a cold, flu, or the coronavirus not often more akin to an accident than a criminal act?^11^ If one cannot prohibit driving because of the possibility of accidents, one cannot quarantine people because they could accidently spread a cold or flu.

Let us take another scenario from driving. What if someone tries to cross a highway on foot or in a wheelchair and is hit? Is the solution to oblige everyone to drive five miles per hour on highways from then on because there exists the possibility that someone in a wheelchair might try to cross and could get hurt? It makes more sense to create a crossing for those who are in danger of being hit, or for them to find safer paths to their destinations. In the case of the corona epidemic, older people with preexisting illnesses can take precautionary measures and isolate themselves if they want to. All these examples show that once one allows aggression against innocent people, such as a lockdown or general quarantine, one is on a slippery slope (Hayek, 2008).

### 3.2 John Stuart Mill on contagious diseases

The more moderate classical liberal Mill (1984 [1871]) arrives at similar conclusion as the radical libertarian Murray Rothbard.^12^ During a parliament hearing in 1871 he gave recommendations for contagious diseases. During the hearing Mill opposes the *Contagious Disease Act*, which allowed policemen to test women suspected to be prostitutes on contagious diseases and confine them in the case they refused to be examined.

Based on his reasoning, one would believe that Mill would also oppose testing and restricting the liberty of people during the corona crisis on the mere possibility that they could be infected. Indeed, Mill states that all judicial rights should hold also in the case of women suspected to be infectious. He even argues that if a woman was voluntarily examined and found to carry the disease, the government should not detain her against her will, because it is not the task of the government to provide security in a preventive way: “I do not think it is part of the business of Government to provide securities beforehand.” (Mill, 1984 [1871], p. 353).

If one applies Mill’s classical liberal reasoning to the corona crisis, one could defend that government should take care of those that get ill but not restrict liberties beforehand. In Mill’s view, someone that acts negligently and infects other (the husband that infects his wife after seeing a prostitute) should be liable for the damage and pay a high penalty. Yet, one cannot make someone liable beforehand. Mill also believes that if no extirpation of the disease is possible, restrictions of liberty cannot be justified. In other words, if “zero‐covid” does not guarantee success, restrictions are unjustified. Furthermore, Mill points out that people do not get infected if they do not expose themselves to the diseases. Similarly, one could argue that in the corona crisis it is possible for the vulnerable to isolate themselves. Whoever wants to take the risk and expose himself or herself to others is free to do so.

In sum, it is interesting that applying the reasoning by classical liberal John Stuart Mill to corona one comes to a similar conclusion applying Rothbardian property rights ethics. Both have in common the principle *in dubio pro libertate*: one cannot restrict liberties without proving that a harm has been done or will be done.

## 4 THE UTILITARIAN ARGUMENT FOR GOVERNMENT RESTRICTIONS IN A PANDEMIC

Let us now address the utilitarian argument in favor of government restrictions of liberty in an epidemic. Classical utilitarianism attempts to maximize the collective interest. No personal interest is greater than the interest of the greatest number (Singer, 2011). Utilitarianism argues that all the consequences of actions, both direct and indirect, short and long term have to be taken into account in decision‐making. Utilitarians aim to maximize what is good for all, defined as the net surplus of what is good for all over what is bad for all. The maximization of the good for all is the only moral principle that is decisions that maximize utility are moral. The idea of utilitarian approaches to the corona crisis is to find “a balance between controlling the pandemic, managing the economic consequences and social costs” (Delanty, 2020, p. 3).

Indeed, proponents of state intervention in the time of the corona pandemic argue that central planning through confinement or other forms of coercion would save lives (Baldwin & Taghipour, 2020; Branswell, 2020; Gavin, 2020; WEF, 2020). The benefits of lockdowns in form of lives saved are annotated on the plus side in their utilitarian considerations. Communities and individuals helping each other in times of distress leading to closer social ties or the subsidies received by governments may be considered additional annotations on the plus side. The argument of the salvation of lives through lockdowns cannot be easily dismissed as it refers to the most important human value. Yet, these utilitarian considerations face important difficulties as Savulescu et al. (2020, p. 630) point out:

The fundamental difficulty facing all of us during this pandemic is that we cannot know for certain which action will be best overall. We do not know what a utilitarian “archangel” would choose: it would require a detailed understanding of the science and facts, the nature of well‐being and an exhaustive understanding of the consequences of our choices.

The utilitarian knowledge problem is related to the problem of economic calculation. The infringements of private property involve (subjective) costs that cannot be calculated and compared to the benefits in a non‐arbitrary way. This is the kernel of von Mises (1920) argument about the impossibility of economic calculation without market prices.^13^

The costs of the COVID‐19 government lockdowns are manifold and unforeseeable. For instance, being confined to one’s own four walls, with the corresponding lack of physical exercise, will lead to increased cardiovascular disease, high blood pressure, diabetes, strokes, cancer, and thromboses, among other things shortening life expectancy (Booth et al., 2007; Lee et al., 2012). Due to lockdowns operations and treatments are postponed, and diseases detected later (Bruno & Rose, 2020; Sikora, 2020). Vaccinations programs are disrupted putting the lives of millions at risk (Hoffman, 2020). Maternal and child mortality will rise possibly causing more than one million deaths in low and middle income countries (Fore, 2020; Roberton et al., 2020).

Moreover, the psychological burden of being locked up is immense. Mousa and Samara (2022) point to the negative impact that the COVID‐19 crisis exerts on the mental health of academics and how meaningful work can mitigate mental health disorders by improving capabilities and maintaining a sense of relatedness. The psychological strain of lockdowns can cause divorces and break up families (Rosner, 2020); traumatization and depression are created (Brooks et al., 2020; Salari et al., 2020; Wu et al., 2020). Domestic violence and child abuse are expected to surge (Usher et al., 2020).

While some people may die due to these infringements of private property; others may be saved. Gorvett (2020) argues that more people will die due to lockdowns than from COVID‐19. But one does not know for sure. Independent from and more fundamental than the question of the net effect on lives of lockdowns is the following problem: How can one compare the costs of the shortened lives due to the lockdowns with the benefits of savings lives of elderly persons? One cannot scientifically compare subjective costs and benefits that affect different persons. Lives, as well as diseases, are *res extra commercium*, they are not traded for money (von Mises, 1998). Consequently, they do not have a market price that would allow for a comparison.

Moreover, the economic havoc created by the lockdown measures is potentially devastating. It can be argued that there would have been an economic crisis anyway due to the distortions created by monetary policy.^14^ The epidemic is only the trigger of the economic crisis. Nevertheless, the crisis is made harsher by the government infringements on private property rights. If people are not allowed to produce, because they cannot leave their homes or open their businesses, production falls.

Business owners who see their lifetime achievement destroyed by the political reaction to the virus could suffer heart attacks, fall into depression, commit suicide (Hawton & Haw, 2013), or become alcoholics. Feelings of shame and failure may affect mental health and increase the risk of depression and suicide (Klofsten et al., 2021). Moreover, the negative psychological consequences of business failure and switching from self‐employment to unemployment are substantial (Nikolova et al., 2021). Especially, small businesses that positively affect productivity and reduce unemployment (Robbins et al., 2000) are negatively affected by lockdowns.

Furthermore, the standard of living will fall as economic activity is suffocated by the confinement. There will be less goods and services available to maintain, let alone improve, quality of life, because these goods and services will simply not be produced. And if the economy of the Western world collapses, the West will buy less goods and services from poor countries. The living standard will therefore also fall in the third world, where it may mean the difference between life and death for many (Fore, 2020). In general, poverty means reduced longevity. Rich people tend to live longer and healthier than poor people (Zaninotto et al., 2020).

We must also consider the possibility of a mass hysteria in an epidemic (Bagus et al., 2021) and an overreaction of politicians toward the threat of an epidemic; including an instrumentalization by politicians posing a threat for democracy (Agamben, 2020). Once fear and anxiety take over and are exploited by politicians for their own personal objectives, the chance of an overreaction and highly invasive interventions increases which cause the aforementioned economic and health havoc without necessity. It is, precisely, private property rights ethically supported that put a limit to such an overreaction. It is a capitalistic system that defends private property rights that inhibits such overreactions. Therefore, in contrary to the critiques that maintain that capitalism is unable to deal with a pandemic, capitalism prevents overreaction and the corresponding damage and harm.

But there are even more adverse long‐term effects of lockdowns. Governments all over the world are advancing on the road to serfdom (Hayek, 2008), controlling their populations and increasing their power relative to the private sector via increased public spending and new regulations. “Centralized management” during the COVID‐19 crisis led to an erosion of basic rights. In many countries, basic democratic rights have been curtailed and freedoms have been interfered with. Mandatory virus vaccination and restrictions on demonstrations are interferences which would have been unthinkable in Western democracies just a few years ago. There is a danger that the restrictions of freedom rights will remain in place in the long term and democracy is being restricted on the pretext of the urgency of a crisis. The restrictions of democracy and liberties during the COVID‐19 crisis could be used as a blueprint for other crises that are perceived as urgent, for example to combat so‐called climate change.

According to the “ratchet effect,” defined by Higgs (1987), government power usually increases in crisis times. However, when the crisis recedes, government power is not reduced to its initial position. Thus, the long‐term victim of the government intrusion may be liberty. More socialist regimes may be instituted. And in these regimes life expectancy is shorter. For instance, the capitalist West Germans had a life expectancy that was about three years longer than that of their socialist East German counterparts (Vogt & Vaupel, 2015).

It is, of course, true that government coercion may increase the life expectancy of some people in the short run. Enforcing confinement in an epidemic is only one example. There are other possibilities. The government may prohibit smoking, or subsidize fruits, vegetables, or sports classes. It may use tax revenue to improve the medical treatments of the population, thereby increasing life expectancy.

Yet, how much artificially increasing of public health is enough? For instance, how much of GDP should be spent on healthcare? Five percent, 10%, 50%, or 90% of GDP? Certainly spending more might increase life expectancy. But how can the government officials know the correct percentage?

Similarly, how much of GDP shall be sacrificed in an epidemic by more or less drastic and long confinement measures? Shall 5%, 10%, 50%, or 90% of productive activities stop in order to slow down the propagation of the virus? There is no non‐arbitrary way for a central planner to decide these matters. All government measures come with costs that cannot be quantified.

Economic calculation in monetary terms is impossible for a government in an epidemic. Instead of calculating in money, the government could calculate with other variables, of course. The government could try to calculate, for instance, in terms of life expectancy, but the result is not clear‐cut at all and would leave aside all other variables, such as costs of psychological diseases or the benefits of enjoying liberty. Moreover, weighing longer life expectancy of some people against the shorter life expectancy of other people would imply that the utility of months to live would be the same for everybody. One month of life for a suffering and ill person may have a different subjective utility than a month of a healthy and free person. In any case, interpersonal utility comparisons are not scientific (Rothbard, 1956). Therefore, all attempts of a government to rationally plan and optimize the outcome of an epidemic with interventions into the liberty of its citizens are arbitrary. The alternative to the arbitrary central planning of government in an epidemic is the libertarian alternative, consisting in the voluntary decisions of private property owners.

## 5 CONCLUSION

This article has analyzed lockdowns from both a property rights perspective as well as a utilitarian perspective and is limited to these approaches. These perspectives have been traditionally used to justify capitalism and the market economy. Naturally, there are many other ethics theories on which one could assess the ethicality of lockdowns such as Kantian virtue ethics, Aristotelian ethics, the ethics of care, Rawlsian justice, or hedonism. These ethical theories are not closely related to the defense of capitalism and may come to different conclusions based on strong arguments. The purpose of our article drawing on Rothbard and J. St. Mill was not to dismiss these arguments. Rather our purpose is limited to show that a capitalist property rights ethics is capable to deal with the challenges of pandemics and comes with important advantages such as the prevention of overreactions. The primary purpose of the paper is to refute the argument that the capitalist system cannot deal appropriately with pandemics. Not only can the ethics of capitalism profitably be employed to pandemics, but it also avoids undesirable effects of government overreaction that we have experienced during the COVID‐19 crisis. We may assume that governments had not much time for consideration and did act in line with their best knowledge and with good intent. Nevertheless, government infringements of private property rights in the COVID‐19 pandemic cannot be justified from a libertarian natural law perspective. They must be considered as violations of universal human rights. Defenders of capitalism argue that a decentralized response to the emergency would be more effective. Every individual, workers and business owners have to decide which risks he would like to take and how far he is willing to reduce social interactions and well‐being. Liberty implies responsibility. At the same time, in a capitalistic market system, entrepreneurial action considers human ends through the anticipation of profits. Unleashing entrepreneurial creativity and genius on every level is more effective than central government planning. The utilitarian argument in favor of government planning fails because economic calculation is not possible. Costs and benefits are immeasurable and subjective. Hence, any decision to bear the costs of the lockdowns remains arbitrary. Governments cannot scientifically balance of costs and benefits of lockdowns. The alternative to state central planning is the libertarian or capitalist approach that give individuals and businesses owners the liberty necessary to deal with an emergency.

## Data Availability

Data sharing is not applicable to this article as no new data were created or analyzed in this study.

## Endnotes

^1^
The centrality of human dignity is typical for Kantian approaches and has been defended in regard to lockdowns by the philosopher Jürgen Habermas (Schwering, 2020) who stated: “the efforts of the state to save every single human life must have absolute priority over a utilitarian offsetting of the undesirable economic costs.”

^2^
It is true, as one referee points out that contemporary capitalism is far away from the libertarian ideal and the two should not be conflated. Indeed, many libertarians consider contemporary societies closer to socialism than capitalism. For our purpose it is important that it is the capitalistic parts in our societies that are criticized for being insufficient to deal with the challenges of the corona crisis. Above mentioned commentators argue that one cannot have full liberty in a pandemic. From their argument it follows that they would regard a libertarian society as even more inadequate to deal with challenges of the corona crisis than contemporary societies which are not fully capitalist.

^3^
For a somewhat different justification of libertarian ethics see Hoppe´s (1988) argumentation ethics or Nozick (2013).

^4^
For an application of the nonaggression principle to controversial cases see Block (1976), Block et al. (2000), Block (2013) or Bagus and de la Horra (2021).

^5^
If people believe that they help themselves by helping others, no coercion would be needed.

^6^
Business owners are responsible for the harm they exert on the property of others such as noise or pollution considering the history of property rights (Rothbard, 1990). If business owners want to open their business in a pandemic, they could put a sign on their property that they are not liable for harm caused by infectious diseases on their property and that customers enter on their own risk.

^7^
On the burden of proof see also Rothbard (1990) who states that conjectures, plausibility or a statistical correlation is not enough to establish proof. The plaintiff must proof strict causality meaning that the defendant caused the violation of the private property rights of the plaintiff beyond any doubts. Mere probability is not enough.

^8^
I owe this challenging example to Mateusz Machaj.

^9^
A lockdown implies the threat of physical violence against those that do not comply. During the corona crisis we saw many instances where the threat of physical violence was carried out, such when policemen chased down and overpowered runners during the lockdown in Spain in 2020.

^10^
There are other opinions on responsibility, such as the one of Hannah Arendt who introduced the concept of universal responsibility––in opposite to guilt. Arendt (1994) argued that “in one form or another all men must assume responsibility for all crimes committed and that all nations share the onus of evil committed by all others.” (p. 131). See also Arendt (2003). The libertarian position, however, is that an individual can only be held responsible for his own voluntary actions and not for crimes committed by other individuals.

^11^
Block (2020, p. 217) seems to suggest that any spread of illness is a rights violation.

^12^
On Mill´s moderate liberalism see Raico (2012).

^13^
On the impossibility of economic calculation in socialism see also von Hayek (1935), Huerta de Soto (2010) and Kavaliou (2018).

^14^
The distortions created by expansionary monetary policy are part and parcel of the Austrian theory of the business cycle. See Hayek (1931), von Mises (1998), Bagus (2007), Bagus (2015), Huerta de Soto (2012).

## References

[beer12431-bib-0001] Agamben . (2020). The invention of an epidemic. https://www.journal‐psychoanalysis.eu/coronavirus‐and‐philosophers/ (original published in Italian on Quodlibet, https://www.quodlibet.it/giorgio‐agamben‐l‐invenzione‐di‐un‐epidemia

[beer12431-bib-0002] Arendt, H. (1994). Organized guilt and universal responsibility. In H. Arendt (Ed.), Essays in understanding 1930–1954 (pp. 121–132). Harcourt, Brace & Co.

[beer12431-bib-0003] Arendt, H. (2003). Collective responsibility. In H. Arendt (Ed.), Responsibility and judgement (pp. 147–158). Schocken Books.

[beer12431-bib-0004] Bagus, P. (2007). Asset prices—An Austrian perspective. Procesos de Mercado: Revista Europea de Economía Política, 4(2), 57–93.

[beer12431-bib-0005] Bagus, P. (2015). The ZIRP trap—The institutionalization of negative real interest rates. Procesos de Mercado: Revista Europea de Economía Política, 12(2), 105–163.

[beer12431-bib-0006] Bagus, P. , & de la Horra, L. P. (2021). An ethical defense of cryptocurrencies. Business Ethics, the Environment & Responsibility, 30(3), 423–431. 10.1111/beer.12344

[beer12431-bib-0007] Bagus, P. , Sanchez‐Bayón, A. , & Peña‐Ramos, J. A. (2021). Covid‐19 and the political economy of mass hysteria. International Journal of Environmental Research and Public Health, 18(4), 1376. 33546144 10.3390/ijerph18041376 PMC7913136

[beer12431-bib-0008] Baldwin, A. , & Taghipour, D. (2020). Why flattening the curve for coronavirus matters. ABCnews. https://abcnews.go.com/Health/flattening‐curve‐coronavirus‐matters/story?id=69519338

[beer12431-bib-0010] Block, W. (1976). Defending the undefendable. Fleet.

[beer12431-bib-0011] Block, W. (2003). The non‐aggression axiom of libertarianism. LewRockwell.com

[beer12431-bib-0012] Block, W. (2013). Defending the undefendable II: Freedom in all realms. Terra Libertas.

[beer12431-bib-0013] Block, W. E. (2020). A libertarian analysis of the COVID‐19 pandemic. Journal of Libertarian Studies, 24(1), 206–237.

[beer12431-bib-0101] Block, W. , Kinsella, S. , & Hoppe, H. (2000). The second paradox of blackmail. Business Ethics Quarterly, 10(3), 593–622. 10.2307/3857894

[beer12431-bib-0015] Booth, F. W. , Laye, M. J. , Lees, S. J. , Rector, R. S. , & Thyfault, J. P. (2007). Reduced physical activity and risk of chronic disease: The biology behind the consequences. European Journal of Applied Physiology, 102(4), 381–390. 17987311 10.1007/s00421-007-0606-5

[beer12431-bib-0017] Branswell, H. (2020). Why ‘flattening the curve’ may be the world’s best bet to slow the coronavirus. Statnews. https://www.statnews.com/2020/03/11/flattening‐curve‐coronavirus/

[beer12431-bib-0018] Brooks, S. K. , Webster, R. K. , Smith, L. E. , Woodland, L. , Wessely, S. , Greenberg, N. , & Rubin, G. J. (2020). The psychological impact of quarantine and how to reduce it: Rapid review of the evidence. The Lancet (British edition), 395(10227), 912–920. 10.1016/S0140-6736(20)30460-8 PMC7158942 32112714

[beer12431-bib-0019] Bruno, B. , & Rose, S. (2020). Patients left behind: Ethical challenges in caring for indirect victims of the Covid‐19 pandemic. The Hastings Center Report, 50(4), 19–23. 10.1002/hast.1168 33448404

[beer12431-bib-0024] Delanty, G. (2020). Six political philosophies in search of a virus: Critical perspectives on the coronavirus pandemic. LEQS Paper No. 156/2020. The London School of Economics and Political Science. https://www.lse.ac.uk/european‐institute/Assets/Documents/LEQS‐Discussion‐Papers/LEQSPaper156.pdf

[beer12431-bib-0025] Fore, H. H. (2020). A wake‐up call: COVID‐19 and its impact on children’s health and wellbeing. The Lancet Global Health, 8(7), e861–e862. 32405458 10.1016/S2214-109X(20)30238-2 PMC7217644

[beer12431-bib-0026] Friedman, D. D. (1973). The machinery of freedom, Harper colophon books. Harper & Row.

[beer12431-bib-0027] Gander, K. (2020, April 8). Is it selfish to go outside, and other ethical questions raised by the COVID‐19 pandemic. Newsweek. https://www.newsweek.com/it‐selfish‐go‐outside‐other‐ethical‐questions‐raised‐covid‐19‐pandemic‐1496572

[beer12431-bib-0028] Gavin, K. (2020). Flattening the curve for COVID‐19: What does it mean and how can you help?. Michigan Health. https://healthblog.uofmhealth.org/wellness‐prevention/flattening‐curve‐for‐covid‐19‐what‐does‐it‐mean‐and‐how‐can‐you‐help

[beer12431-bib-0029] Gorvett, Z. (2020). Why most Covid‐19 deaths won’t be from the virus. BBC.

[beer12431-bib-0030] Grotius, H. (2018). The rights of war and peace: Including the law of nature and of nations. Franklin Classics.

[beer12431-bib-0031] Hawton, K. , & Haw, C. (2013). Economic recession and suicide. BMJ: British Medical Journal, 347(1), f5612. 24046157 10.1136/bmj.f5612

[beer12431-bib-0032] Hayek, F. (1931). Prices and production. Routledge.

[beer12431-bib-0035] Hayek, F. (2008). The road to serfdom. Routledge.

[beer12431-bib-3000] Higgs, R. (1987). Crisis and Leviathan. Oxford University Press.

[beer12431-bib-0036] Hoffman, J. (2020). Polio and measles could surge after disruption of vaccine programs. New York Times Company.

[beer12431-bib-0038] Hoppe, H. (1988). On the ultimate justification of the ethics of private property. Liberty, 2(1), 20–22.

[beer12431-bib-0039] Hoppe, H. (2004). Property, causality, and liability. Quarterly Journal of Austrian Economics, 7(4), 87–95.

[beer12431-bib-0040] Huemer, P. (2020). Libertarian pandemic policy. Fakenous. https://fakenous.net/?p=1439

[beer12431-bib-0041] Huerta de Soto, J. (2010). Socialism, economic calculation and entrepreneurship. Elgar.

[beer12431-bib-0042] Huerta de Soto, J. (2012). Money, bank credit and economic cycles (3rd ed.). Ludwig von Mises Inst.

[beer12431-bib-0043] Kavaliou, A. (2018). Mises' monetary argument in economic calculation debate: Cross the Ts and dot the Is. Procesos de Mercado, 15(1), 95–118.

[beer12431-bib-0044] Klingenberg, E. (2020). Less individualism. In Coronavirus will change the world permanently. Here’s how (U.S. ed.). POLITICO LLC.

[beer12431-bib-0103] Klofsten, M. , MacEachen, E. , & Ståhl, C. (2021). New and small firms in a modern working life: how do we make entrepreneurship healthy? Small Business Economics, 57(2), 755–763.

[beer12431-bib-0045] Kluger, J. (2020). Is ordering takeout unethical? A Medical ethicist answers some of the most common moral questions around coronavirus. Time Magazine. https://time.com/5803980/coronavirus‐ethics/

[beer12431-bib-0046] Koehler, K. (2021). Put that mask over your freedom‐hole, you Covid‐convenient libertarians. The Gauntlet. https://thegauntlet.ca/2021/02/15/put‐that‐mask‐over‐your‐freedom‐hole‐you‐covid‐convenient‐libertarians/

[beer12431-bib-0047] Lee, I. , Shiroma, E. J. , Lobelo, F. , Puska, P. , Blair, S. N. , & Katzmarzyk, P. T. (2012). Effect of physical inactivity on major non‐communicable diseases worldwide: An analysis of burden of disease and life expectancy. The Lancet (British edition), 380(9838), 219–229. 10.1016/S0140-6736(12)61031-9 PMC3645500 22818936

[beer12431-bib-0049] Locke, J. (2018). Two treatises on government. Franklin Classics.

[beer12431-bib-0050] Machan, T. (2002). The right to private property. Hoover Institution.

[beer12431-bib-0051] Masan, L. (2020). Government service regains its catchet. In Coronavirus will change the world permanently. Here’s how (U.S. edition ed.). POLITICO LLC. https://www.politico.com/news/magazine/2020/03/19/coronavirus‐effect‐economy‐life‐society‐analysis‐covid‐135579

[beer12431-bib-0052] McMaken, R. (2015). Imprisoning children in the name of National Security. Mises Institute. https://mises.org/wire/imprisoning‐children‐name‐national‐security

[beer12431-bib-0053] Mill, J. S. (1859). On liberty. John W. Parker & Son.

[beer12431-bib-0054] Mill, J. S. (1984 [1871]). The contagious diseases act. In J. M. Robson (Ed.), The collected works of John Stuart Mill volume XXI—Essays on equality, law, and education (pp. 349–373). Toronto University Press.

[beer12431-bib-0055] Mousa, M. , & Samara, G. (2022). Mental health of business academics within the COVID‐19 era: Can meaningful work help? A qualitative study. Employee Relations: The International Journal (ahead‐of‐print). 10.1108/ER-04-2021-0170

[beer12431-bib-2000] Nikolova, M. , Nikolaev, B. , & Popova, O. (2021). The perceived well‐being and health costs of exiting self‐employment. Small Business Economics, 57, 1819–1836. 10.1007/s11187-020-00374-4

[beer12431-bib-0056] Nozick, R. (2013). Anarchy, state, and utopia. Basic Books.

[beer12431-bib-0057] O´Mara, M. (2020). Big government makes a comeback. In Coronavirus will change the world permanently. Here’s how (U.S. edition ed.). POLITICO LLC. https://www.politico.com/news/magazine/2020/03/19/coronavirus‐effect‐economy‐life‐society‐analysis‐covid‐135579

[beer12431-bib-0058] Olson, W. (2020). Pandemics and power: A notebook. https://www.cato.org/blog/pandemics‐power‐notebook

[beer12431-bib-0059] Oreskes, N. (2020). We need big government to save us from the pandemic. Time Magazine. https://time.com/5823063/we‐need‐big‐government‐pandemic/

[beer12431-bib-0060] Pope Francis . (2020). Fratelli Tutti. Encyclical Letter. https://www.vatican.va/content/francesco/en/encyclicals/documents/papa‐francesco_20201003_enciclica‐fratelli‐tutti.html

[beer12431-bib-0061] Raico, R. (2012). Liberalism: True and false. In Classical liberalism and the Austrian school (pp. 67–110). Ludwig von Mises Institute.

[beer12431-bib-0062] Rand, A. (1967). Capitalism the unknown ideal. New American Library.

[beer12431-bib-2001] Robbins, D. , Pantuosco, L. , Parker, D. , & Fuller, B. (2000). An ampirical assessment of the contribution of small business employment to U.S. state economic performance. Small Business Economics, 15(4), 293–302. 10.1023/A:1011129728483

[beer12431-bib-0063] Roberton, T. , Carter, E. D. , Chou, V. B. , Stegmuller, A. R. , Jackson, B. D. , Tam, Y. , Sawadogo‐Lewis, T. , & Walker, N. (2020). Early estimates of the indirect effects of the COVID‐19 pandemic on maternal and child mortality in low‐income and middle‐income countries: A modelling study. The Lancet Global Health, 8(7), e901–e908. 32405459 10.1016/S2214-109X(20)30229-1 PMC7217645

[beer12431-bib-0064] Rosner, E. (2020). US divorce rates skyrocket amid COVID‐19 pandemic. The New York Post. https://nypost.com/2020/09/01/divorce‐rates‐skyrocket‐in‐u‐s‐amid‐covid‐19/

[beer12431-bib-0065] Rothbard, M. (1990). Law, property rights, and air pollution. In W. Block (Ed.), Economics and the environment: A reconciliation Vol. II (pp. 233–279). The Fraser Institute.

[beer12431-bib-0067] Rothbard, M. N. (Ed.). (1956). Toward a reconstruction of utility and welfare economics. D. Van Nostrand.

[beer12431-bib-0068] Rothbard, M. N. (1982). The ethics of liberty. Humanities Press.

[beer12431-bib-0070] Salari, N. , Hosseinian‐Far, A. , Jalali, R. , Vaisi‐Raygani, A. , Rasoulpoor, S. , Mohammadi, M. , Rasoulpoor, S. , & Khaledi‐Paveh, B. (2020). Prevalence of stress, anxiety, depression among the general population during the COVID‐19 pandemic: A systematic review and meta‐analysis. Globalization and Health, 16(1), 1–57. 32631403 10.1186/s12992-020-00589-w PMC7338126

[beer12431-bib-0084] Salt, S. . (2020). Why lockdowns can halt the spread of COVID‐19. World Economic Forum. https://www.weforum.org/agenda/2020/03/why‐lockdowns‐work‐epidemics‐coronavirus‐covid19/

[beer12431-bib-0071] Savulescu, J. , Persson, I. , & Wilkinson, D. (2020). Utilitarianism and the pandemic. Bioethics, 34(6), 620–632. 32433782 10.1111/bioe.12771 PMC7276855

[beer12431-bib-0072] Schwering, M. (2020). Jürgen Habermas über Corona: “So viel Wissen über unser Nichtwissen gab es noch nie”. Frankfurter Rundschau. https://www.fr.de/kultur/gesellschaft/juergen‐habermas‐coronavirus‐krise‐covid19‐interview‐13642491.html

[beer12431-bib-0073] Shapiro, I. (2020). The government has a lot more emergency powers than libertarians like, but it still can’t control everything.. https://www.cato.org/commentary/government‐has‐lot‐more‐emergency‐powers‐libertarians‐it‐still‐cant‐control‐everything

[beer12431-bib-0074] Sikora, K. (2020). ‘60,000 cancer patients could die because of lack of treatment or diagnosis’: Oncologist on coronavirus dilemma. ITV news.

[beer12431-bib-0075] Singer, P. (2011). Practical ethics (3rd ed.). Cambridge University Press. 39946524

[beer12431-bib-0076] Spencer, H. (1970). Social statics. Robert Schalkenbach Foundation.

[beer12431-bib-0077] Spooner, L. (Ed.). (1973). No treason: The constitution of no authority. Ralph Myles.

[beer12431-bib-0078] Suárez, F. (2012). Tractatus de legibus ac deo legislatore. Editorial CSIC Consejo Superior de Investigaciones Científicas.

[beer12431-bib-0079] The Economist . (2020). The state in the time of Covid‐19. The Economist. https://www.economist.com/leaders/2020/03/26/the‐state‐in‐the‐time‐of‐covid‐19

[beer12431-bib-0080] Usher, K. , Bhullar, N. , Durkin, J. , Gyamfi, N. , & Jackson, D. (2020). Family violence and COVID‐19: Increased vulnerability and reduced options for support. International Journal of Mental Health Nursing, 29(4), 549–552. 32314526 10.1111/inm.12735 PMC7264607

[beer12431-bib-0081] Vogt, T. C. , & Vaupel, J. W. (2015). The importance of regional availability of health care for old age survival—Findings from German reunification. Population Health Metrics, 13(1), 26. 26425117 10.1186/s12963-015-0060-2 PMC4588495

[beer12431-bib-0033] von Hayek, F. (1935). The nature and history of the problem. In Collectivist economic planning (pp. 1–40). Routledge.

[beer12431-bib-0034] von Hayek, F. (1960). The constitution of liberty. University of Chicago Press.

[beer12431-bib-0082] von Mises, L. (1920). Die Unmöglichkeit der Wirtschaftsrechnung im. Archiv für Sozialwissenschaft und Sozialpolitik, 47, 86–121.

[beer12431-bib-0083] von Mises, L. (1998). Human action. A treatise on economics. Mises Institute.

[beer12431-bib-0085] Wu, L. , Guo, X. , Shang, Z. , Sun, Z. , Jia, Y. , Sun, L. , & Liu, W. (2020). China experience from COVID‐19: Mental health in mandatory quarantine zones urgently requires intervention. Psychological Trauma, 12(S1), S3–S5. 32538663 10.1037/tra0000609

[beer12431-bib-0086] Zaninotto, P. , Batty, G. D. , Stenholm, S. , Kawachi, I. , Hyde, M. , Goldberg, M. , Westerlund, H. , Vahtera, J. , & Head, J. (2020). Socioeconomic inequalities in disability‐free life expectancy in older people from England and the United States: A cross‐national population‐based study. The Journals of Gerontology Series A, Biological sciences and medical sciences, 75(5), 906–913. 31940032 10.1093/gerona/glz266 PMC7164527

[beer12431-bib-0100] Žižek, S. (2020). PANDEMIC! COVID‐19 shakes the world. Polity Press.

